# Synthesis and Characterisation of Acrylic Resin-Al Powder Composites Suitable for Additive Manufacturing

**DOI:** 10.3390/polym12081642

**Published:** 2020-07-23

**Authors:** J. J. Relinque, Ismael Romero-Ocaña, Francisco J. Navas-Martos, F. J. Delgado, M. Domínguez, S. I. Molina

**Affiliations:** 1 Departamento de Ciencia de los Materiales e I. M. y Q. I., IMEYMAT, Facultad de Ciencias, Universidad de Cádiz, Campus Río San Pedro s/n, 11510 Puerto Real (Cádiz), Spain; fjavier.delgado@uca.es (F.J.D.); sergio.molina@uca.es (S.I.M.); 2 Centro Tecnológico del Plástico ANDALTEC, Ampliación Polígono Cañada de la Fuente, C/Vílchez s/n, 23600 Martos (Jaén), Spain; francisco-javier.navas@andaltec.org; 3 Departamento de Física de la Materia Condensada, IMEYMAT, Facultad de Ciencias, Universidad de Cádiz, Campus Río San Pedro s/n, 11510 Puerto Real (Cádiz), Spain; manolo.dominguez@uca.es

**Keywords:** polymer–matrix composites, stereolithography, physical methods of analysis, mechanical properties, thermal properties

## Abstract

Stereolithography is an additive manufacturing technology commonly used to build either prototypes or final parts. Nevertheless, the manufacture of structural parts has been ruled out owing to the poor mechanical properties of conventional UV-curable resins. Moreover, the inventory of available commercial resins is still limited and they exhibit low thermal and electrical conductivity values. In this work, some composite materials were designed using Al microparticles dispersed within an SLA commercial resin matrix. These composites overcame the difficulties caused by the light scattering effect during the photopolymerisation process in the SLA technology. Dispersion of the filler was characterised by means of SEM/EDX and AFM. The composites exhibited improved thermal and mechanical behaviour in comparison with the pristine resin. The simplicity of the synthesis method used to prepare the composites provides a convenient starting point to explore new ways of designing composites for SLA with improved mechanical and functional properties.

## 1. Introduction

Traditionally, the methodology used in the manufacturing industry to build any object involved the use of expensive moulds or tooling to fabricate each part (e.g., injection moulding or machining technologies). In recent years, 3D printing or additive manufacturing (AM) techniques have emerged as processing methods with technological applications in the framework of industry 4.0 [1,2,3,4,5,6]. AM techniques are based on a manufacturing concept that is depositing sequentially layers of material until the part is complete [7]. Nowadays, 3D printing techniques are present in several industrial fields, for instance, the aeronautical industry [8], aerospace [9,10], naval [11], medical [12,13], or automotive industries [3,14]. It is perceptible that AM techniques mark a turning point for the manufacturing industry. The advantages of AM techniques are countless, among which, the following are highlighted [1,5,15,16]:

- Costs’ reduction when compared with traditional manufacturing techniques because less raw material is required.
- Eco-friendly character as less raw materials and energy are required in comparison with conventional manufacturing.
- Completely computer-driven processes. Operators are only required at certain stages of the process with increasing automation.
- Freedom of design allowing to overcome the design restriction and the geometry limitations in conventional manufacturing.

Within this framework, several kinds of AM techniques have broken into the market including selective laser sintering/melting, fused filament fabrication, and stereolithography (SLA) among others. The differences between these techniques lies in the type of material used and how the AM system works. SLA was developed in the 1980s by 3D Systems (Valencia, CA, USA) [17]. It is a vat photopolymerisation technique in which a laser beam is focused through the transparent vat, which contains a photocurable resin, inducing a polymerisation reaction and, eventually, transforming the liquid resin into a solid object with a previously designed 3D geometry. This building concept is one of the most versatile AM techniques as it allows to obtain thinner laminate layers, which leads to producing parts with low porosity [18]. Despite being limited by many factors such as poor mechanical properties [18], one of the highlighting applications of SLA lies in the field of tissue engineering applications [19,20]. For this reason, SLA has recently attracted research interest in obtaining composite materials with improved properties compared with pristine photocurable resins [21,22].

Great efforts are currently under way in terms of reinforcing photocurable resins in order to improve their mechanical and functional properties. On the one hand, it was reported in several studies that the incorporation of additives to pristine resins, even in low percentages, can modify tensile strength, Young modulus, flexural strength, fracture toughness, and hardness significantly [23]. In this sense, the shape, size, and other properties of the additive play a decisive role in the modification of these properties [24,25]. On the other hand, the dispersed particles can scatter the light, and thus the penetration of the UV light can be reduced by affecting the processability [26]. Indeed, this was one of the glitches found during the present work. Generally, one of the main problems of conventional manufacturing technologies is the use of expensive moulds that are usually made of metals parts whose continuous use during the manufacturing process requires fast heating/cooling cycles. As cooling is a very time-consuming step in the manufacturing process and depends exclusively on the properties of the materials, cheaper and more suitable composite moulds with improved thermal conductivity properties could reduce the costs and help to decrease production times [27].

The present study reports on the design of composite materials based on the dispersion of Al microparticles within a commercial acrylic resin (AR) suitable for processing in an SLA system. The main objective of this experimental study was to improve the thermal conductivity of the pristine resin by the preparation of UV-curable resin with different loads of Al powder. The selection of Al was based on its excellent thermal conductivity and it was reported in the literature as an appropriated additive for UV-curable resins in terms of processability. In addition, the manufactured composites were characterised in order to determine structural modifications caused by the dispersion of Al additive, as well as the influence on mechanical and thermal properties.

## 2. Materials and Methods

The AR-Al composites were prepared upon a commercial acrylic resin, namely *FLGPCL02* supplied by the company *Formlabs* and microparticulate Al powder (averaged equivalent diameter ca. 12 μm supplied by *Feroca*, Madrid, Spain). In accordance with the supplier, the resin is a mixture containing methacrylated oligomers and monomers and an unknown photoinitiator.

The synthesis of the composites was carried out by means of paddle stirring, specifically using a rod finished in an anchor as recommended for high viscous fluids such as the AR [28,29,30,31]. AR and Al were added together to a beaker and underwent stirring for 165 min at 850 rpm, keeping the beaker isolated from light in order to prevent the start of the polymerisation reaction during the dispersion of the Al particles within the AR. The stirrer employed was a *VOS 40* supplied by *VWR*. Composites were prepared increasing Al wt. % from 5 to 30%, according to Table 1.

Upon concluded the stirring, the homogeneous mixture was immediately poured into the vat of an SLA machine (*Form 1+* supplied by *Formlabs*, Somerville, MA, USA), without a break in continuity, and test specimens were printed with a layer precision of 0.1 mm. The printer employs an EN 60825-1:2007 Class 1 Laser (405 nm, violet laser, 250 mW). The amount of laser power and curing duration is predetermined for *FLGPCL02* resin. After processing, 2-propanol (supplied by *VWR Chemicals*, Radnor, PA, USA) was used to wash the samples.

The specimens mentioned above were used to evaluate the mechanical and thermal properties as well as for structural determinations. In this way, different specimens were manufactured by means of SLA technology according to UNE-EN-ISO 527, UNE-EN-ISO 180, UNE-EN-ISO 75, and UNE-EN-ISO 306 standards. These specimens were intended respectively for the tensile tests (1BA specimens tested with a Universal Testing Machine *Tinius Olsen 10KS*, Horsham, PA, USA, at 1 mm/min, properties as an average of five experiments), Izod impact testing (ISO 180 U specimens tested with a *Metrotec IMPats 15* equipment, Lezo, Spain, hammer with 5 J of nominal energy impact, strength as an average of no less than five experiments), and HDT and Vicat temperature tests (ISO 180 U specimens tested with an *Astfaar MP-3*, apparatus, Milan, Italy, at 120 °C/h, 250 g loading for the HDT experiments and 50 °C/h, 5000 g loading for the Vicat experiments; HDT, and Vicat temperatures as an average of three experiments, respectively). Furthermore, specimens for thermal conductivity measurement (cylindrical specimens tested with a *TA Instruments DTC-25* conductivimeter, New Castle, DE, USA, accuracy of 3%) were manufactured by SLA according to ASTM E1530 standard. Determination of glass transition temperatures, curing enthalpies and degradation temperatures of the different composites was performed by means of DSC with a *Mettler Toledo Star^e^ 1 System*, Columbus, OH, USA. DSC curves were analysed by means of *STAR^e^* software of the own apparatus. Experiments were carried out under 10 °C/min heating ramps in nitrogen atmosphere. Thermal stability was studied by means of TGA with a *TA Instruments Q50* thermogravimetric analyser. Experiments were performed under 10 °C/min heating ramps in nitrogen atmosphere. DSC and TGA/DTG curves were analysed triply to obtain the above-mentioned thermal properties.

Samples for structural determinations under SEM/EDX and AFM were prepared according to the following procedure. From a cylindrical specimen, a thin sheet of material was cut off using a *Struers Accutom-5* disc cutter, Ballerup, Denmark. The so obtained sheet was ground and polished in a *Struers LaboPol-5* machine using a *Struers DP-Paste*, *M* diamond paste. After these two steps, the sample was flattened, reducing its roughness as much as possible in order to facilitate contact under AFM observation. The polished sheets were sonicated in water to clean up the surface and then fixed to appropriate sample holders. Subsequent observations were performed using an *FEI Nova NanoSEM 450* SEM/EDX apparatus, Hillsboro, OR, USA, and a *Veeco Instruments MultiMode NanoScope IIIa* AFM, Plainview (NY), USA, equipped with a scanning thermal microscopy (SThM) system from *Anasys Instruments*, Santa Barbara (CA), USA, capable of providing thermal conductivity contrast images of areas within composite materials. Furthermore, AFM images were processed and corrected from artifacts using *Gwyddion* software (Brno, Czech Republic) [32]. Mean roughness determinations were also estimated by means of *Gwyddion*.

## 3. Results and Discussion

### 3.1. Structural and Compositional Characterisation

**Figure 1:**
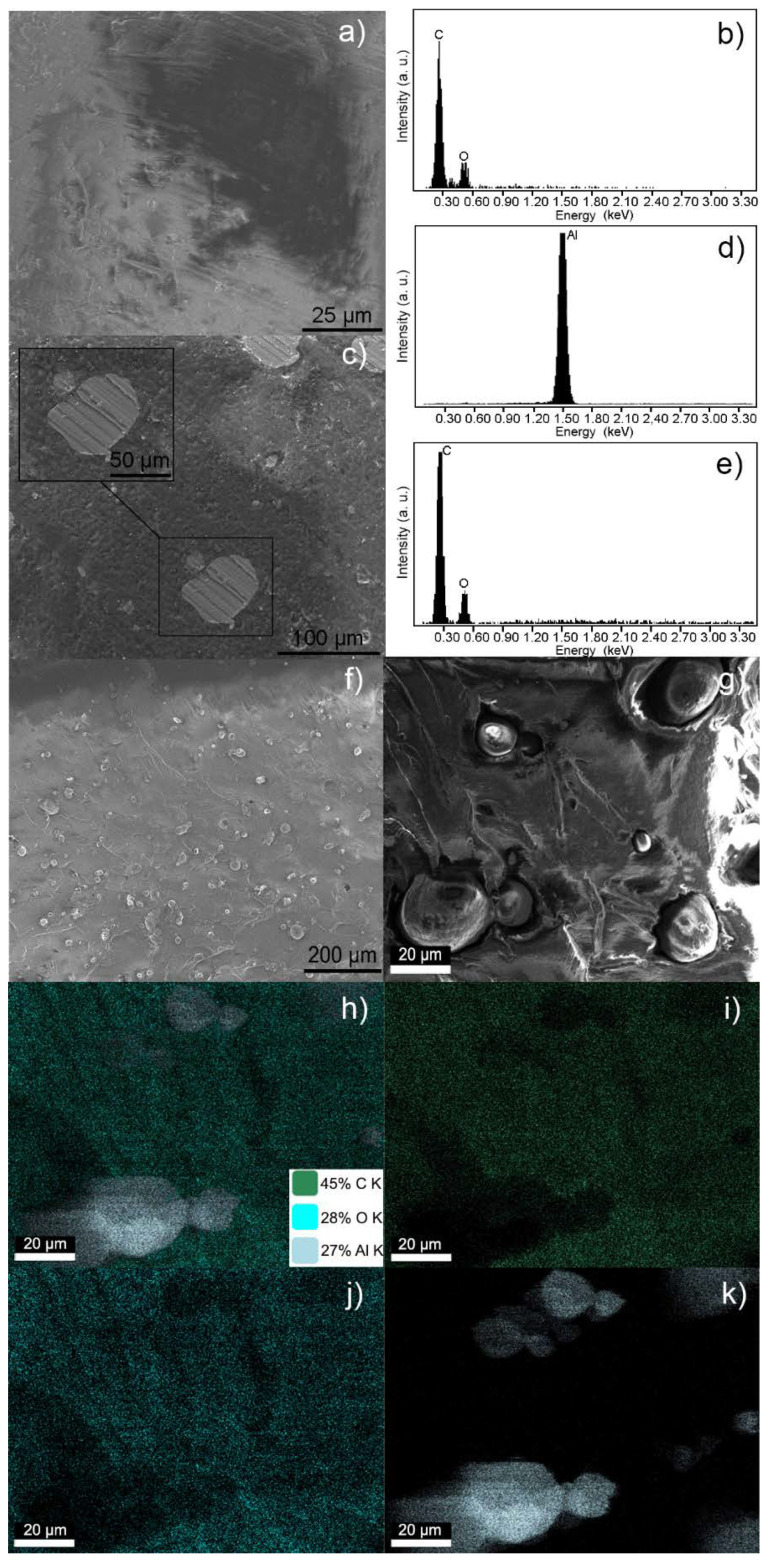
(**a**) SEM image and (**b**) its associated EDX spectrum of an acrylic resin (AR) sample. (**c**) SEM image of AR-Al15, showing in more detail a particle of interest located in the frame, and the EDX spectra associated with (**d**) the particle of interest and (**e**) the particle-free zone. (**f**) General SEM image of AR-Al30; (**g**) enlargement of the EDX mapping area; (**h**) global EDX map with estimation of the elementary composition in wt. %; and EDX maps of (**i**) C composition, (**j**) O composition, and (**k**) Al composition.

The samples of the different composites (Table 1), obtained from the circular specimens previously manufactured by SLA, were analysed by means of SEM/EDX and AFM. As with thermoplastics, resins are susceptible to deterioration during the observation process under an electron source. In fact, their low electrical conductivity makes them tend to accumulate negative charges on the surface that, among other factors, hinder their analysis by electron microscopy [33]. Therefore, analyses were carried out on selected compounds, including the pristine resin, an intermediate loaded compound (AR-Al15), and the sample with the highest percentage of Al in the composite series (AR-Al30). Figure 1a–e shows the SEM images and the EDX spectra obtained from AR and AR-Al15 samples, while SEM images and EDX maps corresponding to AR-Al30 are depicted in Figure 1f–k.

In the case of the pristine resin (Figure 1a), the SEM/EDX analyses were performed in localised points and the resulting images elucidated that AR samples presented a rough surface. Striations were also observed, which may be owing to the preparation process to get the suitable specimens for AFM and SEM/EDX (the same kind of specimen was used for both tests). On the one hand, the EDX spectrum (Figure 1b) revealed the presence of C and O, as expected for a resin based on an acrylic polymer. On the other hand, EDX did not show the presence of N, which led to ruling out nitrogen precursors in the commercial acrylic resin used.

The image corresponding to the AR-Al15 composite (Figure 1c) showed a clearly distinguishable particle dispersion, mainly owing to the different textures between the areas with Al and the pristine resin. By enlarging the areas where these presumed Al particles were localised, smooth and homogeneous surfaces were observed in contrast to the rough surfaces noticed in the pristine resin (but for the aforementioned striations). EDX spectra allowed to confirm the Al composition of the particles observed in the SEM image (Figure 1d) by comparison with spectra performed in particle-free zones (Figure 1e).

The structure corresponding to the composite AR-Al30 allowed to correlate the increase in load with a greater number of the observed Al particles, as depicted in Figure 1f. Thus, with a material loaded with conductive particles, the SEM examination was simpler and EDX mapping was able to be performed to characterise the composition of a complete area of the samples instead of the localised points previously analysed in samples loaded with a lower percentage of Al. The aforementioned EDX mapping was carried out in the specific area presented in Figure 1g. As a result, several particles with a similar morphology to those identified as Al in the previous analysis performed in the AR-Al15 samples were observed. Therefore, it is reasonable to identify them with the Al particles. Indeed, the EDX maps (Figure 1h–k) confirmed the presence of Al distributed in those areas, which coincides with the location of those particles previously supposed to be metallic. Variable compositions were found in terms of C and O distributed throughout the sample area, with a particularly low concentration of them in those areas where the Al particles are localised. Of particular interest is the data of Al concentration, 27 wt. %, that was obtained in the analysis, because of presenting a value reasonably close to the nominal composition of the composite (30 wt. %).

The results obtained by EDX mapping did not determine a significant composition of O in the locations where the presence of Al particles were identified, which means that no oxidation took place. In the specific case of O, as it is generally part of the polymer matrix composition, the presence of this element in the analysed samples can be attributed both to the environmental contamination, which could have been deposited on the surface of the sample, as well as to the polymer chemical composition.

The characterisation by SEM/EDX allowed to define the structure of AR-Al composites, which consisted of Al particles more or less homogeneously distributed (to a greater or lesser extent, depending on the Al load) within the acrylic resin. Increasing concentrations of Al seemed to minimise surface negative charge accumulation, thus facilitating SEM/EDX observations. This may be owing to a better electrostatic dissipation induced by the Al dispersion.

**Figure 2:**
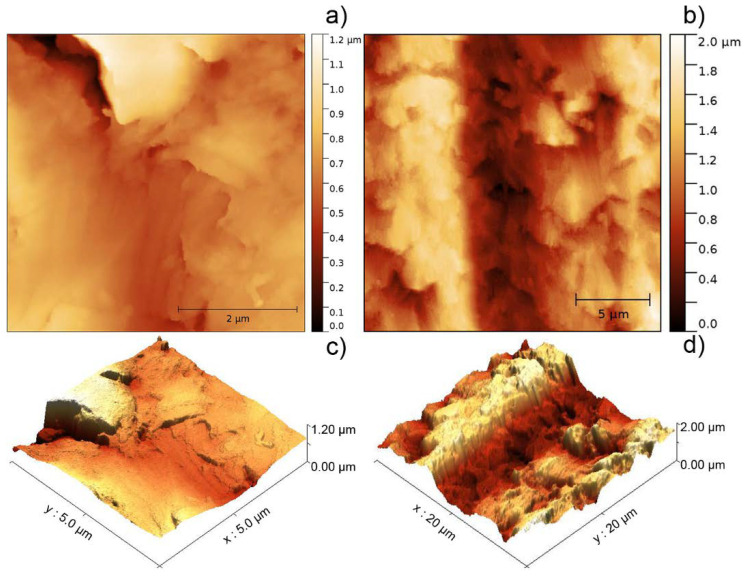
Topographic analysis of the polymer matrix of the samples of (**a**) pristine resin and (**b**) composite AR-Al30. 3D images (**c**,**d**) correspond to the topographic images (**a**,**b**), respectively.

A study of the samples was also carried out using AFM in order to confirm the aforementioned structure of the composites. The AFM analysis allowed to get more detailed information about the surface of the composites determining particles that were effectively exposed. A topographic comparison between two surfaces attributed to the resin is shown in Figure 2; the surface of a sample of pristine resin (Figure 2a) and the surface of a sample in an area supposed to be metal-free of the AR-Al30 composite (Figure 2b).

In general, the topography of the samples showed profiles not too steep for AR-Al composites, preventing the occasional loss of contact between the sample and the probe of the AFM equipment and allowing the acquisition of good surface images. In the particular case of the pristine resin (Figure 2a), the observed surface was more homogeneous in terms of height than the AR-Al30 sample, whose surface was analysed in a metal-free zone area with the aim of determining the effect that the percentage of Al particles exerted on the resin topography. Finally, a remarkable difference in roughness was found, and it was attributed to the different hardness of those materials present in the composite. This is consistent with that observed in the SEM images (Figure 1a), where the surface of the pristine resin is less rough than those observed in the composites AR-Al15 (Figure 1c) and AR-Al30 (Figure 1f). The estimated mean roughness for pristine resin (110.3 nm) and for the composite AR-Al30 (328.9 nm) confirm the textural discussion.

**Figure 3:**
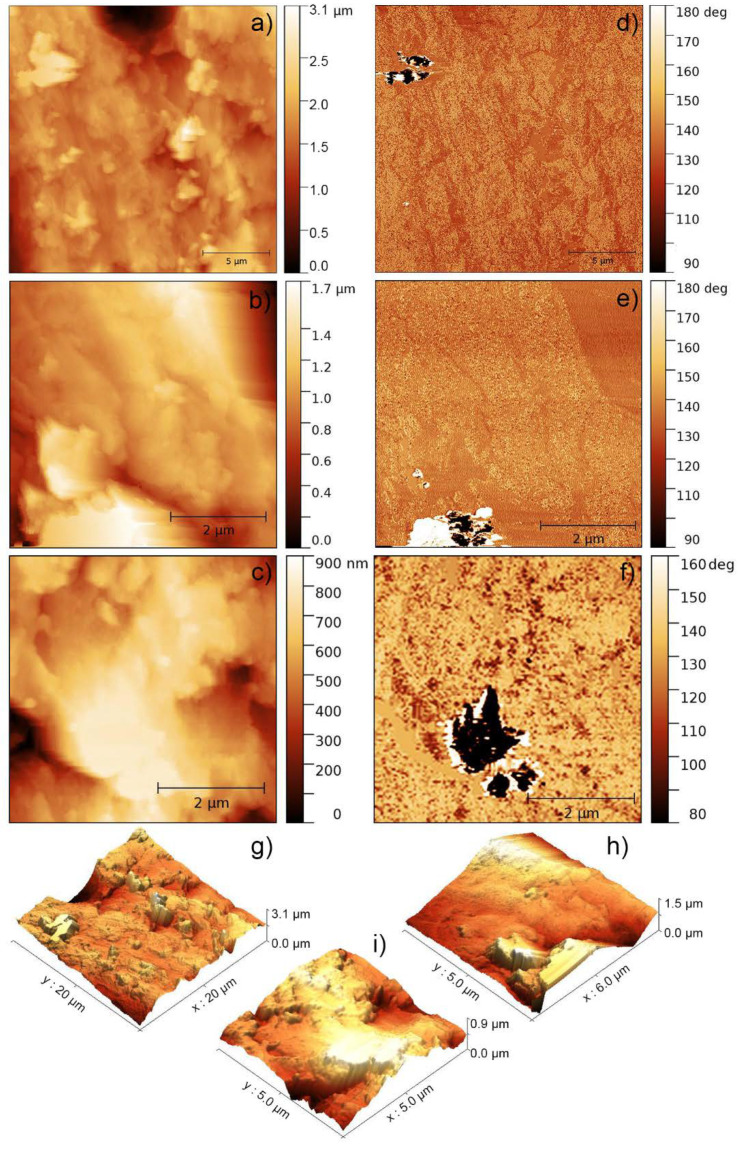
AFM characterisation of AR-Al30 samples. (**a**–**c**) Topographic images of different areas of the sample containing Al particles. (**d**–**f**) Corresponding phase contrast images and (**g**–**i**) their respective 3D reconstructions.

In Figure 3, the AFM analysis of several particles of an AR-Al30 composite sample specifically carried out in those zones where Al particles were present is depicted. The characterisation of the AR-Al30 particles presented great difficulties as a result of the loss of contact between probe and sample, owing not only to the topography, but also to a highly variable mechanical interaction of the two materials present in the composite. Indeed, several particles were observed whose images are not presented in this work owing to their low quality as a result of the intense noise in the transitions between the metal particles and the resin during surface scanning. This fact is a new point that, added to all those discussed earlier in this paper, helps to determine the presence of dispersed particles in the polymer matrix. Figure 3 shows the best images that were taken of some of the metallic particles dispersed in the AR-Al30 composite, sorted by decreasing slope of the topographic profile, that is, the most rugged topographies with less obvious phase contrasts are shown first. 

In the topography images (Figure 3a–c), regions especially high (lighter in colour) were detected that could be attributed to the surface of the Al particles present in the acrylic resin. The 3D reconstructions (Figure 3g–i) allowed to improve the visualisation of the aforementioned regions and to identify them as higher formations compared with the surrounding resin. It also reinforces the previously outlined hypothesis that the feature of the sample profile is owing to the preparation method. In addition, the confirmation of the presence of different materials in the sample was again provided by obtaining phase contrast images. In fact, the phase contrast images (Figure 3d–f) corresponding to the topographic images exhibited contrasts undoubtedly related to the Al particles introduced as fillers in the composites. Considering that the obtained phase contrasts cannot be directly related to topographic features and that the particles observed in topography do not exactly correspond to the phase contrasts, it can be concluded that the observed particles only protrude partially from the surface, in the proportion shown by the phase contrasts.

**Figure 4:**
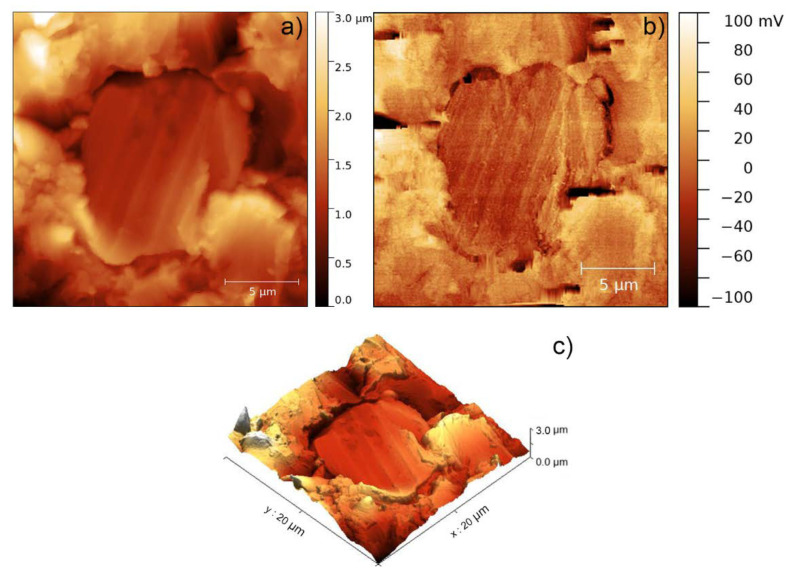
AFM characterisation of AR-Al15. (**a**) Topography of a region containing a conductive particle, (**b**) corresponding thermal image obtained by scanning thermal microscopy (SThM), and (**c**) 3D reconstruction of the sample.

The use of SThM mode in AFM was impossible owing to both the instability of the tapping contact mode and the strong dependence of the thermal mode on said stability. For this reason, it was decided to make observations on those composite samples with a lower Al percentage in order to verify if a lower dispersion of particles could be related to a greater ease to achieve a more stable contact. In this sense, Figure 4 shows the characterisation of a particle contained in a sample of the AR-Al15 composite. In the topography of the Figure 4a, it was distinguished a feature that may be associated to an Al particle exposed on the surface. The stability of the contact allowed to carry out a scan in SThM mode, obtaining the corresponding thermal image (Figure 4b).

In this mode, voltage measurements correspond to the voltage across a Wheatstone bridge in which the resistive AFM tip corresponds to one of the branches. Before the tip comes into contact with the sample surface, the Wheatstone bridge is balanced using a variable resistor located at the branch of the bridge in front of the resistive AFM tip. When the tip comes into contact with the sample, as current is flowing through the resistor tip and the heat produced by the Joule effect is being dissipated, this heat flows through the sample and the temperature of the tip goes down. The higher the thermal conductivity of the region of the sample below the tip, the lower the temperature of the tip. As a consequence of this change in the tip temperature, its resistance changes and the voltage across the Wheatstone bridge changes too. Thus, the temperature of the probe can be correlated to the thermal conductivity of the sample, so that the darkest region presented in Figure 4b (which coincides with the exposed surface attributed to an aluminium particle) corresponds to an area where the temperature measured by the probe is lower, and therefore, the thermal conductivity of the sample surface is higher, as expected of a metal particle compared with the polymer matrix. The three-dimensional reconstruction shown in Figure 4c helped the particle visualisation and defined it as an especially smooth surface in comparison with the surrounding resin.

As a summary of the structural characterisation of AR-Al composites, it has been possible to determine that these composites consist of a dispersion of Al particles through the polymer matrix. SEM/EDX and AFM have determined the structural and compositional nature referred to and have allowed to know both topographic and textural details of the different samples in a fully concordant complementary study.

### 3.2. Mechanical Characterisation

**Figure 5:**
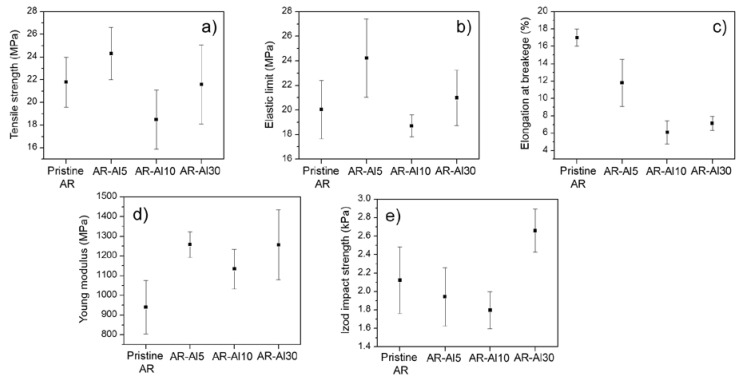
Data obtained from tensile and Izod tests on AR-Al composites: (**a**) tensile strength, (**b**) elastic limit, (**c**) elongation at break, (**d**) Young modulus, and (**e**) and impact strength, with confidence intervals.

The mechanical characterisation of the different AR-Al composites was carried out by performing both tensile and Izod impact tests on those samples manufactured by means of SLA from the different Al-liquid resin solutions. In Figure 5, the mechanical properties of the composites were compared to those exhibited by the pristine resin.

When it comes to polymer composites, the introduction of fillers or additives may lead to the increase of some mechanical properties to the detriment of others. In the particular case of the materials used in this work, it was expected that elastic properties such as Young modulus or the elastic limit were improved, while plastic properties such as elongation at break or tensile strength may worsen in variable levels [34,35,36]. Therefore, an optimal solution may be to reach a balance between the percentage of Al microparticles and the improvement–deterioration ratio of mechanical properties. In addition, the maximum percentage of Al microparticles that liquid resin can admit without losing their processability by SLA must also be taken into consideration.

AR-Al composites’ behaviour was consistent with the general behaviour aforementioned. Figure 5a,b shows that variations in the strength at break and the elastic limit values were subtle and overlapping confidence intervals of pristine resin and several composites. On the one hand, not surprisingly, a decrease in the elongation at break was observed as the Al loading increased (Figure 5c). More specifically, the elongation at break exhibited by the AR-Al30 composite (highest Al load in the series) was 58% lower than that shown by the pristine resin. On the other hand, Young modulus of AR-Al30 composite improved that of the pristine resin by 34% (Figure 5d). Those AR-Al composites with intermediate Al loadings also presented lower values of elongation at break and higher values of Young modulus, respectively, without following a clear trend.

As expected, the absorbed energy in the Izod tests (Figure 5e) followed a decreasing trend as the Al loading in the sample increased, although the energies absorbed by the AR-Al5 and AR-Al10 composites kept within the confidence interval of the values obtained for the pristine resin. The AR-Al30 composite presented anomalous behaviour, showing the highest impact energy absorption in the series. In general, the absorbed energy values by the tested materials did not exhibit great variability.

It is important to remark that the analysis of mechanical properties was performed on specimens manufactured by SLA without any post-curing process (green specimens), so that the variable behaviour of the composites in terms of mechanical properties could be related to the degree of cure, which could have been affected by the introduction of the Al microparticles. In order to clarify this point, DSC curves of the materials were obtained and the results are exhibited in the next section, where the present discussion is extended in terms of the thermal behaviour of the composite materials.

The mechanical properties assessed in this work were compared with other studies published in the literature. Gurr et al. (2008) carried out the formulation of photocurable acrylic resins with dispersed silica nanoparticles and evaluated their mechanical properties in terms of Young modulus and toughness, obtaining variable results for both properties as the percentage of silica nanoparticles increased [37]. Gurr et al. (2010) also carried out the formulation of two photocurable acrylic resins containing hybrid calcium phosphate nanoparticles and laminated silicates, reaching increases in Young modulus lower than those obtained in this work and even in some cases with values within the confidence intervals of the pristine resin and, therefore, without a clear trend [38]. Taormina et al. (2018) carried out another study based on the in situ growth of silver nanoparticles (AgNP) during the SLA process using a photocurable acrylic resin specifically formulated for that work, obtaining variable values of Young modulus and tensile strength for a series of composites with an increasing percentage of AgNP particles [39]. Sciancalepore et al. (2017) confirmed a poor improvement in tensile strength and a small drop in the elongation at breakage of the AgNP nanocomposites aforementioned, with values within the confidence interval of the pristine resin [40]. Therefore, it leads us to conclude that composites and nanocomposites based on photocurable acrylic resins did not experience a substantial improvement in mechanical properties. In the specific case of the composites studied in this work, the loading of Al microparticles did not mean a dramatic worsening of the elongation at break and, on the contrary, significantly improved Young modulus without substantially modifying the elastic limit and tensile strength.

### 3.3. Thermal Characterisation

Thermal characterisation of the different AR-Al composites was carried out through DSC curves, softening temperature (T_Vicat_), and heat deflection temperature (T_HDT_). Thermal stability of the composites was studied by TGA/DTG. In addition, thermal conductivity was also determined in order to verify if the percolation threshold was exceeded. The DSC curves obtained are available in Figure S1 (given as Supplementary Material).

The DSC curves showed subtle thermal changes located between 50 and 60 °C, which can be associated to the glass transition phenomenon, and exothermic peaks ranging from 155 to 165 °C, which should be ascribed to the curing process. The heating ramp ended up causing the degradation of the composites at temperatures ca. 300 °C. Resins’ chemical structure is that of thermoset polymers consisting of a cross-linked network of polymer chains. Thermosets typically present a glass transition linked to the amorphous nature of the material structure and crystallisation peaks associated to the crystallinity that certain regions of the polymer chains can acquire during the heating process once the material passes from a vitreous to a viscoelastic state prior to reaching the melting point. Melting of thermoplastics supposes the state of greatest disintegration and mobility of their polymer chains, forming a quasi-liquid material [41]. By contrast, when it comes to thermosets, the crosslinking of their polymer chains limits their ability to flow, whereby glass transition takes place at higher temperature values and no peak corresponding to the fusion process is observed. In addition, because the glass transition is a phenomenon associated with the amorphous part of the polymers, the higher the structural order, the more complex it is to observe the phenomenon. Indeed, this transition would only be observed in those parts of uncured material fully integrated into the polymer matrix. Thermosets tend to show glass transitions over a wide range of temperatures and the methodology to be used for their determination is usually more demanding [41,42].

The chemical structure of thermosets is susceptible to evolution and variability during a thermal process, as the curing process can be initiated in various ways (including heat and photopolymerisation) and extended over time depending on the established conditions. It is also common for the post-processing stage to include subjecting the parts manufactured by photopolymerisation to irradiation sources under controlled conditions. When it comes to thermostable DSC curves, the literature indicates that it is possible to observe exothermic peaks that would correspond to a cure favoured by the increase in temperature of the sample during said study. These peaks would decrease or even disappear when the degree of cure is increased by the application of some post-curing process of successive DSC cycles to the sample [39,43,44].

The DSC curves of the AR-Al composites verify the discussion previously exposed. Glass transitions were so subtle that it was necessary to magnify the curves in the specific region. Other works have been found where similar situations occurred [42]. According to the results, the trend followed by the obtained T_g_ values is to increase with the percentage of Al, which is also described in the literature for different composite systems [45,46].

The enthalpy associated with the curing peaks (ΔH_c_) was determined by performing numerical integration of the exothermic peaks observed in the DSC curves (Figure S1) in order to characterise the degree of curing of the composites. The peaks were located over the temperature range between 155 and 165 °C, being the lower one the corresponding to the pristine resin (155 °C). Al particles dispersed into the polymer matrix may have introduced a subtle shift effect on the temperature value from which the curing process begins. In any case, enthalpy values show a decreasing trend as the percentage of Al increases up to reach 30 wt. %. For this composite, an upturn in released heat during the curing process is observed that occurs as a result of the heating experienced by the sample during the DSC test. Enthalpy values are directly related to the degree of cure of the sample. As green parts were studied in this work, which were not subjected to a post-curing process after their manufacturing, an evolution of crosslinking in the sample during the time until testing was expected. Additionally, adding a thermal conductive material into the polymer matrix may have improved the curing process during the sample examination by means of increasing heat transfer through the composite materials, as the curing enthalpies observed for AR-Al5 and AR-Al10 composites were lower than those shown by the pristine resin. In the specific case of the AR-Al30 composite, particles’ dispersion may have not been as effective as in that of those composites with lower Al loading. Indeed, 30 wt. % was considered as the operational limit of the SLA printing of the composites designed in this study, as severe surface defects were observed in AR-Al30 specimens. As the reinforcement of elastic properties is strongly dependent in the dispersion of the fillers [47], at a filling rate of 30 wt. % of Al, the formation of agglomerates is higher (as observed in SEM) and, consequently, the UV penetration impairs.

Considering the results of some of the mechanical properties previously discussed (Figure 5), particularly the Izod impact resistance (Figure 5e), the AR-Al30 composite tends to improve on average to the rest of materials. Samples that are not submitted to a post-curing process normally present a lower degree of cure; contain a higher percentage of monomers and oligomers that remain unreacted; and, therefore, have less consolidated crosslinking. This is also consistent with improved mechanical properties in terms of plasticity and poorer mechanical properties in terms of elasticity [48]. The DSC curves seem to correlate lower cure enthalpies with higher stiffness and less elongation, closer to those of the pristine resin.

**Figure 6:**
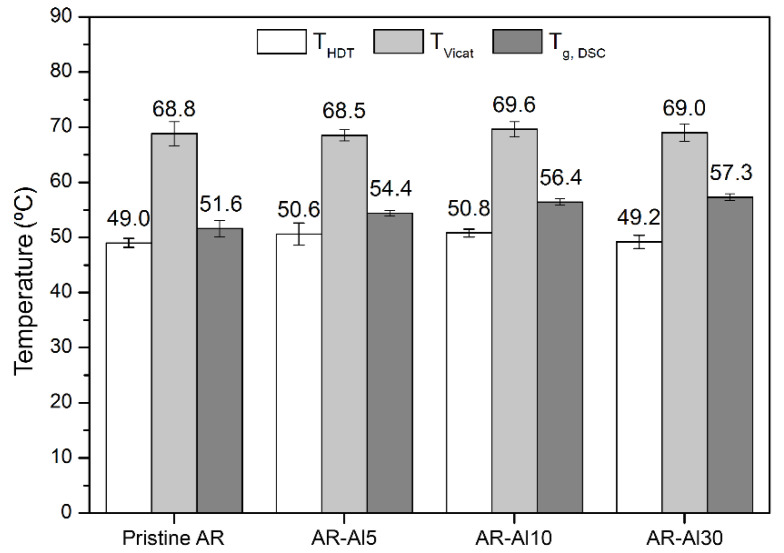
Comparison of T_g_, T_HDT_, and T_Vicat_ (with confidence intervals) obtained for the different AR-Al composites.

In the case of thermoplastic materials, T_Vicat_ and T_HDT_ are usually reasonably close to T_g_. This is because, despite not representing the same phenomenon, they have certain analogy as softening is a consequence of the glass transition [49]. However, as discussed above, thermosets generally present a more subtle vitreous transition that occurs over a wider temperature range. Therefore, their softening temperatures are not necessarily similar to their glass transition temperatures and may be located between T_g_ and the degradation temperature [49]. Differently, T_HDT_ is more variable because it depends on multiple factors such as the load applied to the sample, the efficiency of the dispersion of filler particles into the matrix, the granulometry and aspect ratio of the particles, thermal hysteresis of the composite, and so on. Although formally, an increase in T_HDT_ might be expected when T_g_ increases [50], there are also examples in the literature where T_HDT_ decreases against expectations [51]. Figure 6 shows a comparison between the three thermal parameters T_HDT_, T_Vicat_, and T_g_. On the one hand, T_g_ showed an increasing trend with the Al percentage and presented lower values than T_Vicat_. On the other hand, T_HDT_ and T_Vicat_ did not display any dependence on the Al loading because all the analysed composites showed similar values. In the particular case of T_HDT_, it remained close to T_g_, being only slightly lower.

**Figure 7:**
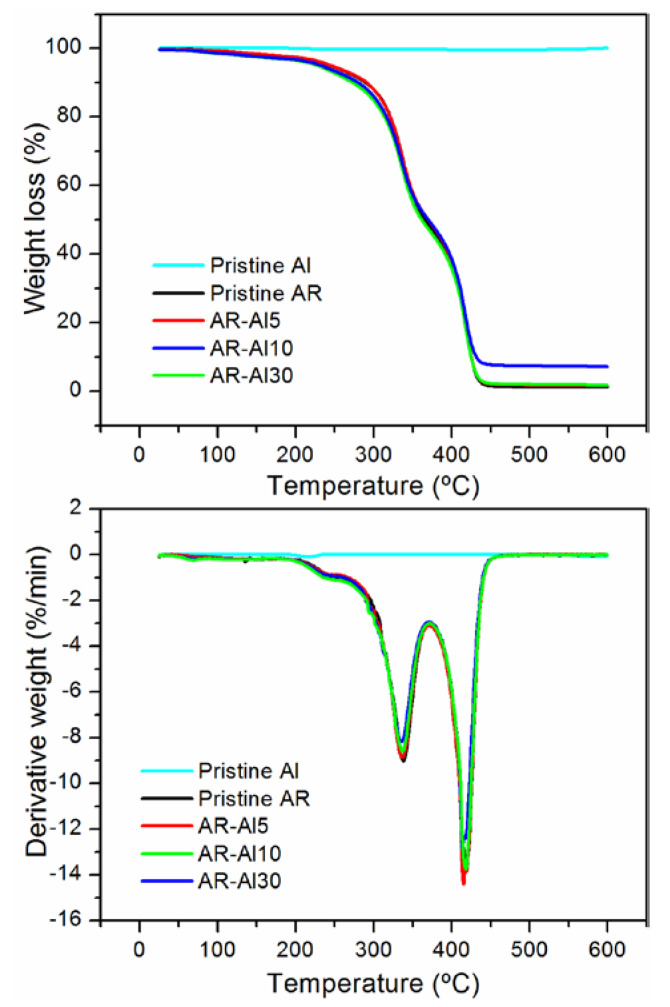
TGA (upper graph) and DTG (lower graph) curves of AR-Al composites. The curves correspond to the average of three different samples.

Thermal stability of the AR-Al composites was assessed through TGA/DTG. The curves corresponding to the different composites and pristine resin and the filler are depicted in Figure 7. The temperatures corresponding to the peaks of maximum weight loss, T_max1_ and T_max2_, are listed in Table 2.

For acrylic resins, the literature points out two stages of maximum weight loss. The first stage, ranging from 280 to 375 °C, would be associated with the decomposition of vinyl groups at the end of polymer chains, whereas the second one could be ascribed to random C–C scissions through all over the polymer structure [52]. With respect to acrylic resin-based composites, many works report on different trends observed for the maximums when introducing fillers to the pristine resin. Depending on the geometry of the filler, and the extent of dispersion and adhesion both peaks could shift to higher temperatures owing to the prevention of volatile polymeric material diffusion induced by the fillers [52,53,54]. Notwithstanding, in some cases, maximums would remain invariable [53] or even decrease [55] owing to chemical interactions chain-filler.

Concerning the results of the present work, two degradation stages were identified, as reported. The weight loss maximums for the composites were included within the confidence intervals of those corresponding to the pristine resin, or slightly decreased (T_max1_ of AR-Al30). In view of these results, it can be concluded that no strong polymer–metal adhesion arose from filling the pristine resin, which could be in good agreement with the variable mechanical properties observed. Nevertheless, the composites did not significantly impair the thermal stability of the pristine resin.

**Figure 8:**
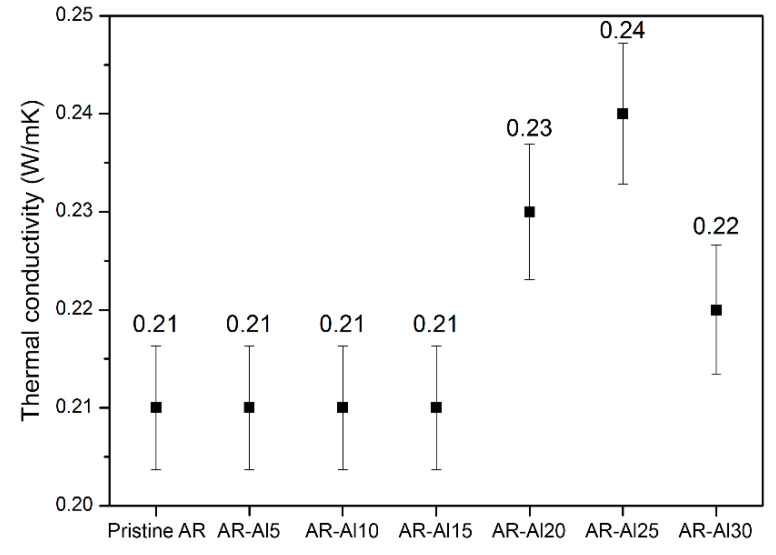
Thermal conductivities (with confidence intervals) obtained for AR-Al composites.

Finally, measurements of the thermal conductivity of the AR-Al composites were carried out. Thermal conductivity was determined through a standardised test from circular specimens prepared by means of SLA printing and the results are depicted in Figure 8. According to these obtained results, the addition of Al did not induce any improvement in the thermal conductivity of the composites with respect to pristine resin (0.21 W/mK) before reaching 20 wt. % of Al particles. The maximum thermal conductivity was found in the AR-Al25 composite (0.24 W/mK). Nevertheless, the increase in thermal conductivity only occurred in those composites with an Al loading lower than 30 wt. %, as the AR-Al30 composite showed a decrease in conductivity. Considering that the samples of the AR-Al30 composite showed surface failures, it was clarified that an excessive percentage of Al could negatively affect the photopolymerisation during the manufacturing process of the specimens. In fact, the previously discussed values of some mechanical and thermal properties seemed to be out of the expected trend for AR-Al30 composite and the thermal conductivity results appear to be consistent with it.

## 4. Conclusions

A simple synthesis methodology was conceived to obtain suitable composite materials to be used in the manufacture of parts using SLA, a 3D printing technology. Suspensions of Al powder in a commercial acrylic resin could be satisfactorily stabilised and used to obtain test specimens of composite materials at different loadings of the metallic powder. Dispersion of Al was confirmed by means of SEM/EDX. A methodology for the observation of composite samples under AFM/SThM was developed and the results of this characterisation were consistent with the previous results.

The combined assessment of structure and mechanical and thermal properties allowed to establish 25 wt. % as the maximum Al load to obtain parts without defects. Furthermore, the mechanical properties did not experience a decay (as is usual in this kind of composite materials) and, concerning thermal properties, the glass transition was improved, thus implying better thermal stability, and an incipient growth of thermal conductivity was observed.

The characterisation proposed in this work, which includes the combined use of microstructural SEM/EDX measurements, mechanical properties determination (tensile and impact testing), topographical measurements by AFM, and thermal characterisation (DSC, TGA/DTG, HDT/Vicat, thermal conductivity, and nanoscale measurements by SThM), has been successfully applied to assist in the development of composites for SLA consisting of metal microparticle dispersed acrylic resins.

Future work should be devoted to enhance percolation networks within the composite using conductive nanomaterials (metallic nanoparticles or graphene-like nanofillers) that may allow to reduce additive loads and, eventually, improve the conductivity of SLA printed parts.

## Figures and Tables

**Table 1:** Prepared composites. AR, acrylic resin.

Composite	Filler	Concentration (wt. %)
Pristine AR	-	-
AR-Al5	Al	5
AR-Al10	Al	10
AR-Al15	Al	15
AR-Al20	Al	20
AR-Al25	Al	25
AR-Al30	Al	30

**Table 2:** Temperatures at maximum weight losses (T_max1_ and T_max2_) deducted from TGA/DTG analysis.

Composite	T_max1_ (°C)	T_max2_ (°C)
Pristine Al	-	-
Pristine AR	338.8 ± 0.6	418.6 ± 2.3
AR-Al5	336.6 ± 1.2	416.1 ± 1.9
AR-Al10	337.3 ± 1.0	418.1 ± 0.8
AR-Al30	335.7 ± 0.5	416.5 ± 2.1

## Supplementary Materials

The following are available online at https://www.mdpi.com/2073-4360/12/8/1642/s1, Figure S1: DSC curves of AR-Al composites. The upper right inset shows a zoom of the range of temperature where glass transition temperatures are located. The upper left inset shows the enthalpy values corresponding to curing peaks.
