# Supplementary material for: Synthesis and Characterisation of Acrylic Resin-Al Powder Composites Suitable for Additive Manufacturing
Source: Polymers (Basel). 2020 Jul 23;12(8):1642. doi: 10.3390/polym12081642 (PMC7465903; doi:10.3390/polym12081642)
Supplement: Supplementary file 1 [file polymers-12-01642-s001.pdf]

Supplementary Materials

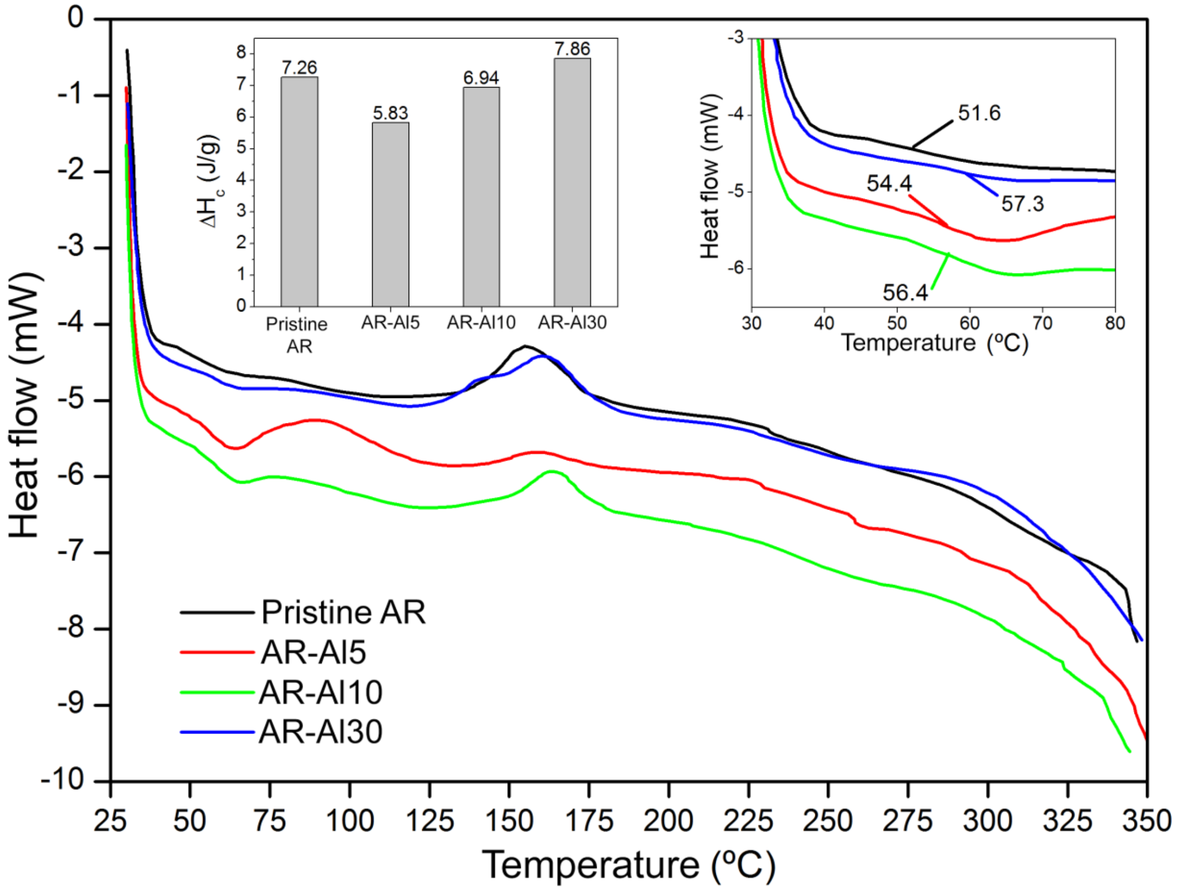

Figure S1: DSC curves of AR-Al composites. The upper right inset shows a zoom of the range of temperature where glass transition temperatures are located. The upper left inset shows the enthalpy values corresponding to curing peaks.
